# Evaluation of Venous Congestion Using Beside Ultrasonography by the Nephrology Consultant: The VExUS Nexus

**DOI:** 10.24908/pocus.v7iKidney.15341

**Published:** 2022-02-01

**Authors:** Abhilash Koratala

**Affiliations:** 1 Division of Nephrology, Medical College of Wisconsin Milwaukee, Wisconsin USA

**Keywords:** VExUS, POCUS, venous Doppler, point of care ultrasound, heart failure

## Abstract

In patients with heart failure and cardiorenal syndrome, lingering congestion is associated with worse outcomes. As such, titrating diuretic or ultrafiltration therapy based on objective assessment of volume status plays a crucial role in the management of these patients. Conventional physical examination findings and parameters such as daily weight measurement are not always reliable in this setting. Recently, point of care ultrasonography (POCUS) has emerged as an attractive enhancement to bedside clinical examination in assessing fluid volume status. Specifically, Doppler ultrasound of the major abdominal veins gives additional information about end-organ congestion when used in conjunction with inferior vena cava ultrasound. Moreover, these Doppler waveforms can be monitored in real time to gauge the efficacy of decongestive therapy. Herein, we present a case that illustrates the utility of POCUS in the management of a patient with heart failure exacerbation.

## Case

A 62-year-old man with a history of diabetes mellitus type 2, coronary artery disease, heart failure with reduced left ventricular ejection fraction (approximately 20%), and stage 3 chronic kidney disease was admitted to the hospital for management of right lower extremity ulceration possibly requiring an above-knee amputation. His home medications were resumed including the loop diuretic, bumetanide 2mg twice a day. Serum creatinine at presentation was 2.4 mg/dL (baseline ~1.6 - 1.8), which increased to 3.2 mg/dL over the next 2 days. Nephrology was consulted for acute kidney injury. The documented weight was 66.8 kg, which decreased by 6 kg compared to that of previous day. Notably, the weighing equipment was different as the patient was transferred to a different ward. Cardiopulmonary examination was unremarkable except for basal crackles and mild jugular venous distension. There was trace pedal edema on the left and right leg showed the known ulceration with surrounding inflammation. The patient was a poor historian and endorsed ‘feeling fine’ without the need for supplemental oxygen. We were asked if the patient was ‘overdiuresed’ based on the reduction in weight accompanied by rise in serum creatinine. 

**Figure 1:**
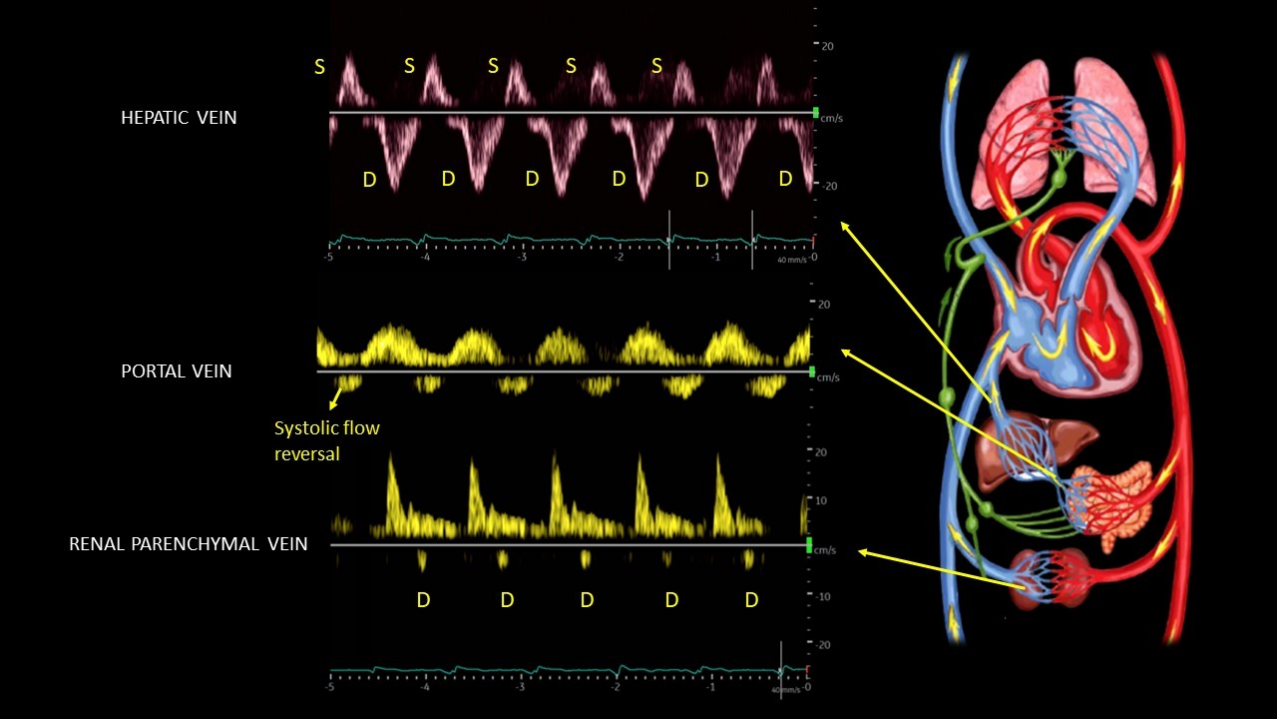
Venous Doppler images at the time of initial POCUS scan demonstrating severe congestive pattern in hepatic, portal, and renal parenchymal veins. There is flow reversal during systole (flow away from the heart) in all the three veins. S = systolic wave; D = diastolic wave. Hemodynamic circuit illustration licensed fromshutterstock®.

**Figure 2:**
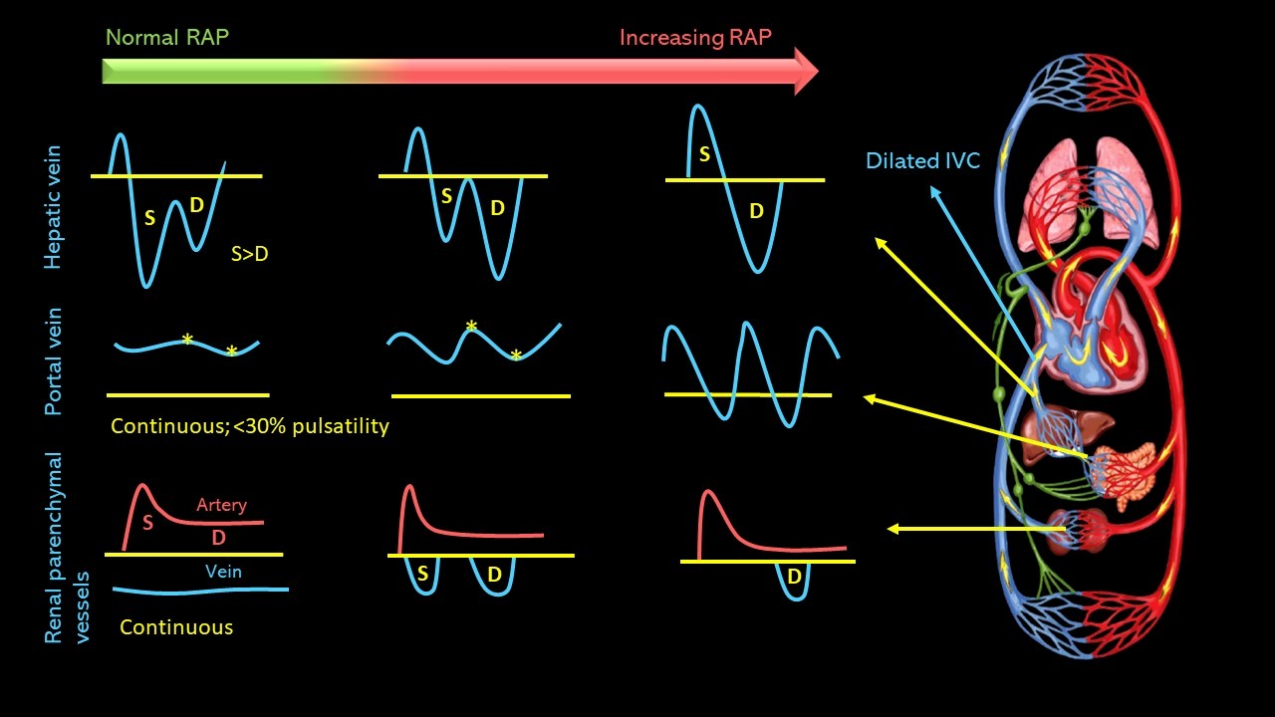
Illustration showing the natural history of venous Doppler waveforms with increasing right atrial pressure. Dilatation of the inferior vena cava (typically >2 cm) usually precedes these changes. S = systolic wave, D = diastolic wave. Asterisks on the portal vein waveform indicate the highest and lowest amplitudes during a given cardiac cycle used to calculate percentpulsatility. Note that the normal portal vein waveform is above the baseline and the renal is below, which reflects the direction of blood flow towards and away from the transducer, respectively.

**Figure 3:**
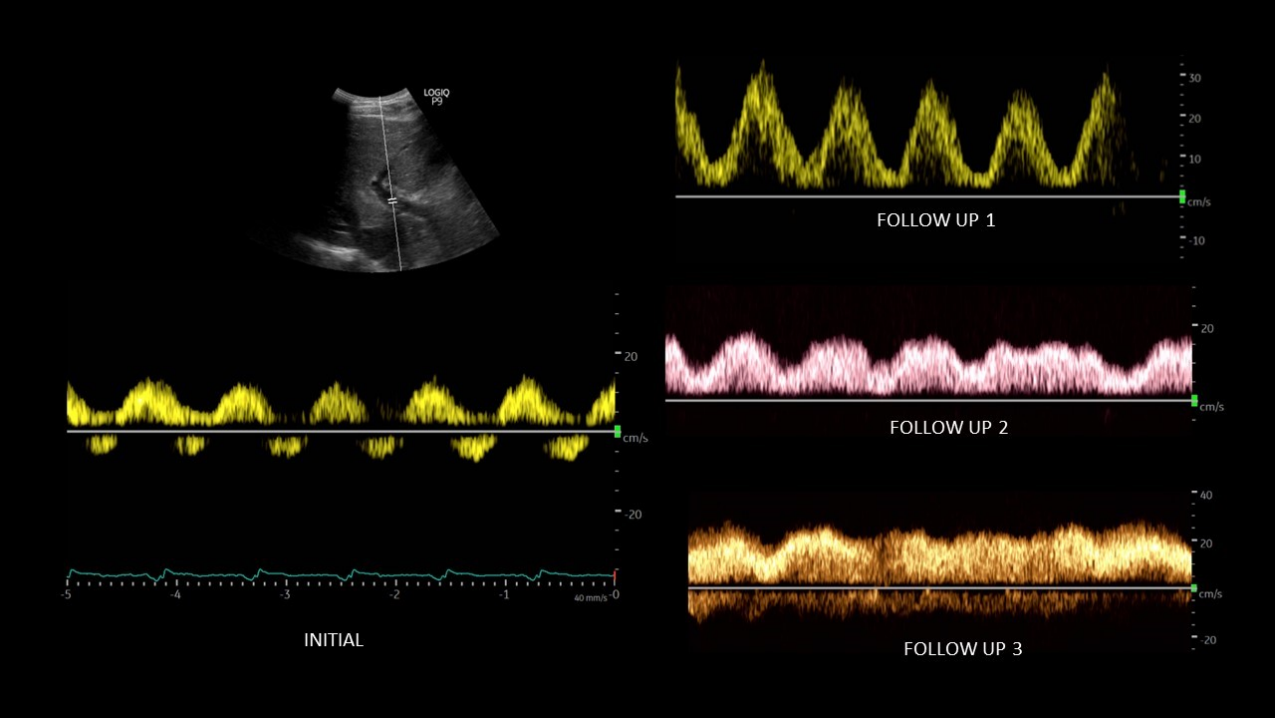
Changes in portal vein waveform in response to decongestive therapy. Note that the systolic flow reversal has disappeared initially followed by gradual decrease inpulsatility.

**Figure 4:**
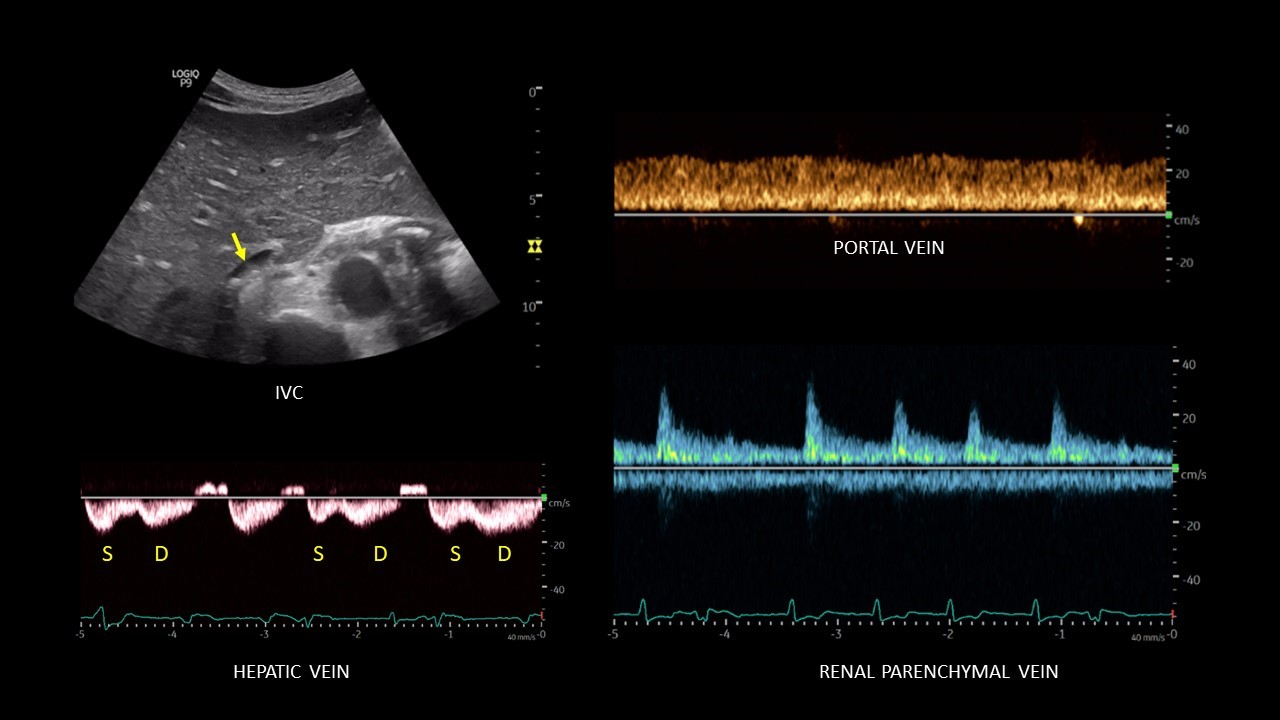
Small inferior vena cava and normalized venous Doppler waveforms following decongestive therapy. Hepatic waveform shows both S and D components below the baseline in most cardiac cyclesand portal and renal waveforms are continuous. Note that the patient had atrial fibrillation at the time of this scan, which decreases the amplitude of S wave.

A point of care ultrasound (POCUS) examination was performed to elucidate the fluid volume status and guide management. Focused echocardiogram was notable for reduced left ventricular systolic function with global hypokinesis, moderate right ventricular enlargement without obvious interventricular septal flattening, and trace tricuspid regurgitation. There was trace pericardial effusion. Inferior vena cava (IVC) was dilated with a maximal diameter of ~2.5 cm and minimal respiratory variation consistent with a right atrial pressure of at least 15 mmHg. Lung ultrasound demonstrated 3 or more B-lines per rib-interspace in the examined zones bilaterally. A venous excess ultrasound (VExUS) grading was performed at the same time. Hepatic vein spectral Doppler waveform revealed only diastolic (D) wave below the baseline with systolic (S) flow reversal. Portal vein Doppler revealed 100% pulsatility with flow reversal during systole and intra-renal waveform showed flow only during diastole, i.e., only D-wave below the baseline (Figure 1). Taken together, these findings were consistent with severe systemic venous congestion as well as pulmonary congestion in the setting of heart failure exacerbation. Figure 2 illustrates the normal Doppler waveforms and their transformation with elevated right atrial pressure. The diuretic therapy was intensified, and the serum creatinine improved to 2.1 mg/dL during the next 4 days. The portal vein waveform was almost normalized during this period (Figure 3), though hepatic and intra-renal vein continued to show congestive pattern albeit with subtle changes suggesting improvement in right atrial pressures. For example, amplitude of the systolic flow reversal has reduced in the hepatic vein and systolic flow started to appear below the baseline in the intra-renal vein. IVC remained >2 cm in diameter but the degree of inspiratory collapse has improved. However, the serum creatinine worsened to 2.7 mg/dL 2 days later, and at the same time, he underwent right above-knee amputation. Intra-operatively, he developed forward failure requiring ionotropic therapy and subsequently developed oliguric renal failure requiring continuous renal replacement therapy. Inotropes were weaned off in one day and mechanical fluid removal was continued. Pre-operative rise in creatinine despite improving congestion was likely secondary to cardiac pump failure. On post-operative day 10 and prior to discharge, a repeat POCUS examination showed a small collapsible IVC as well as normalization of all the venous Doppler waveforms (online Video S1, Figure 4), indicating the resolution of the venous congestion. In addition, lung ultrasound demonstrated A-line pattern in the sono-auscultated zones.

The take-home points from this case file are: 

Doppler ultrasound of the hepatic, portal, and intra-renal veins can be used as an adjunct to inferior vena cava ultrasound and other parameters such as body weight to assess venous congestion in patients with heart failure. Having a simultaneous electrocardiographic tracing helps with accurate interpretation of the waveforms by delineating the phases of cardiac cycle.

These venous waveforms reflect the end-organ congestion and in fact, have shown to outperform isolated central venous pressure (CVP) in predicting the risk of acute kidney injury in selected patients 1.

VExUS scans can be used to monitor the efficacy of decongestive therapy in heart failure patients. As demonstrated in this case, portal vein waveform typically improves the other waveforms as well as the IVC distension likely because of the ‘partial’ transmission of CVP to hepatic sinusoids due to the resistance offered by hepatic venous sphincters 2.

If the serum creatinine worsens despite improvement in the venous waveforms, impairment of the forward flow should be suspected and managed accordingly. On a similar note, caution must be exercised in patients with chronic pulmonary hypertension, where overzealous attempts to normalize all the waveforms may lead to decreased preload and consequent pump failure. Having a baseline VExUS grade helps in such cases, if available.

## Conflict of Interest

The author declares that no conflict of interest exists.

## Consent

Informed consent has been obtained from the patient for the publication of this case study.

## Supplementary Material

 Video S1 Video S1. Transverse axis ultrasound of the inferior vena cava (IVC) at the time of initial evaluation compared to that of post-decongestive therapy. Note how it transformed from a ‘round’ vessel with minimal respiratory variation to a collapsed slit-like structure.
